# Psychological distress of Ghanaian couples after unsuccessful treatment for infertility

**DOI:** 10.4314/gmj.v57i4.4

**Published:** 2023-12

**Authors:** Stephen M Arhin, Kwesi B Mensah, Evans K Agbeno, Felix Yirdong, Kwame Opoku-Agyeman, Charles Ansah

**Affiliations:** 1 Department of Pharmacology, Kwame Nkrumah University of Science and Technology (KNUST), Ghana; 2 Department of Obstetrics and Gynaecology, School of Medical Sciences, University of Cape Coast, Ghana; 3 Department of Psychological Medicine and Mental Health, University of Cape Coast, Ghana; 4 Department of Psychology, The New School, New York, U.S.A; 5 Department of Pharmacology, School of Medical Sciences, University of Cape Coast, Ghana

**Keywords:** Infertility, subfertility, drug treatment, depression, anxiety, stress, psychological distress, Ghana

## Abstract

**Objective:**

The main objective of the study was to assess psychological distress and to identify any gender specific differences in the psychological distress among infertile couples after one year of unsuccessful pharmacotherapy.

**Design:**

A descriptive cross-sectional study.

**Setting:**

The study was conducted in four fertility clinics in the Cape Coast Metropolis.

**Participants:**

One hundred and twenty respondents (71 women and 49 men) were recruited by simple random sampling.

**Statistical analysis:**

Statistical analysis was done using SPSS (v. 25). Psychological distress scores were presented as Mean±SD and were analysed using One-way ANOVA, followed by Bonferroni's post hoc test. Associations between exposures and outcomes were measured using relative risk.

**Outcome measure:**

The main outcome measure was the level of depression, anxiety, and stress among infertile couples after unsuccessful pharmacotherapy.

**Results:**

Anxiety was the predominant psychological distress experienced by respondents (60.8%), followed by depression (43.3%) and stress (37.5%). Generally, psychological distress scores increased with age among female respondents but decreased with age for male respondents. The duration of infertility only significantly affected anxiety (*p*=0.01) but not depression (*p*=0.51) and stress (*p*=0.06) levels. Approximately 31.7% of respondents reported experiencing extremely severe anxiety. Male respondents reported higher degree of depressive symptoms than females (46.9 vs. 40.8%).

**Conclusion:**

Unsuccessful pharmacotherapy of infertility is associated with varied degrees of psychological distress among Ghanaian infertile couples, which can be affected by age, duration of infertility and gender.

**Funding:**

None declared

## Introduction

Infertility remains a major public health issue as it affects approximately 12-15% of couples worldwide.^1^ In Ghana, the prevalence rate is estimated to be between 12-16%.^2^ ^3^ Higher prevalence rates, 25-30%, have been reported in some parts of sub-Saharan Africa.^4^ Although various treatment options exist, pharmacotherapy remains the major first-line treatment, especially in resource-constrained or deprived settings where access to assisted reproductive technology is limited.^5^

The diagnosis of infertility, and anticipations associated with treatment outcomes may predispose couples to varied psychological distress symptoms.

Epidemiological evidence shows that infertile couples experience emotional and psychological distress, including depression, anxiety, stress, social isolation, reduced self-esteem, inferiority complex and poor interpersonal relationships.^6^

In sub-Saharan Africa, particularly Ghana, children are highly esteemed such that a delay in childbearing and voluntary infertility is stigmatized.^7^,^8^ The sociocultural pressures, anticipatory side effects of medications, potential treatment failure, and financial commitment are significant contributory factors to the psychological distress experienced by infertile couples.^9^,^10^

Infertility related psychological distress could manifest as diminished sexual satisfaction, marital displeasure, poor communication, marital instability and somatization.^11^ Although psychological distress could affect treatment outcomes, fertility specialists typically focus on medical therapies, while the psychological issues go unnoticed or untreated.

The prevalence of depression, anxiety and stress among infertile couples varies among cultures worldwide.^12^ Even though female-associated factors contribute only about one-third of the causes of infertility, nonetheless, in many African societies, women are erroneously blamed for a couple's struggle with fertility.^12^ It has been suggested by some authors that women experience more infertility-associated psychological distress as compared to men.^13^ Indeed, in Ghana, most of the existing studies have examined the psychological impact of infertility on women, with little or no focus on their male partners.^14^ ^15^ Majority of Ghanaian infertile women (64%) reported feeling stigmatized and this perceived stigmatization was associated with increased psychological distress.^3^ Alhassan, et al.^12^ reported that depression among infertile Ghanaian women was 62%. Among Ghanaian women, the authors further reported a positive correlation between depression, age and duration of infertility. Similarly, Oti-Boadi and Oppong Asante 16 reported a negative correlation between psychological distress, somatization and negative religious coping strategies in Ghanaian women. The prevalence, gender disparity and the extent to which these psychological distresses impact the quality of life, compliance and therapeutic outcomes in males in Ghana is unknown.

It has been reported that only 20% of Infertile couples achieve success with pharmacotherapy in Ghana.^17^ Both men and women exhibit significant levels of anxiety after one year of unsuccessful infertility treatment. 18 Due to low socio-economic levels and the cost of infertility treatment, any unsuccessful infertility treatment creates an additional financial burden for couples which could potentially exacerbate their psychological distress. Therefore, the objective of this study was to examine the levels of depression, anxiety and stress among infertile couples after one year of unsuccessful drug treatment, and to evaluate the role of gender in these psychological symptoms' experienced.

## Methods

### Study design

A descriptive cross-sectional study, conducted at four different fertility clinics in the Cape Coast Metropolis (Central Region-Ghana), over a two-year period, (March 2019 to February 2021) to assess psychological distress associated with unsuccessful infertility treatment among couples undergoing pharmacotherapy for infertility.

### Participants

A total of 482 infertile couples were initially recruited to assess the effectiveness of pharmacotherapy. These clients were followed up for a period of 12 months to assess pregnancy outcomes. A total of 120 respondents (71 women and 49 men) with unsuccessful treatment outcomes were enrolled on the study using a simple random sampling technique. Infertility was defined as the inability of sexually active couples to conceive after one year of unprotected penovaginal sexual intercourse (and a period of six months if the woman was above 35 years of age).^19^ Treatment was defined as unsuccessful when either one or both partners managed for infertility did not see any effect with regard to conception over a period of at least one year.

### Inclusion and exclusion criteria

To be eligible for the study, respondents should:

1. have been diagnosed with primary infertility (i.e not conceived before), or secondary infertility (i.e conceived or given birth before but has unwanted interval).
2. not have had conception after one year of pursuing drug treatment.
3. be without any diagnosed mental disorders or not receiving any treatment in this area
4. be willing to participate in the study.

Excluded from the study were:

1. patients who achieved clinical pregnancies (females).
2. defaulters.
3. patients with a history of mental disorder prior to infertility diagnosis.

### Data collection

Standardized psychological distress questionnaires were administered to respondents to obtain responses related to their psychological state. In addition, socio-demographic information including age, gender, occupation, educational level, religion, and duration of infertility, were obtained from the respondents. Each item on the scale was explained to respondents in their preferred language.

### Assessing the psychological status of respondents

The Depression, Anxiety, and Stress-Scale (DASS-21) developed by Lovibond and Lovibond was used in this study. 20 The instrument has been validated to be sensitive and reliable in assessing the severity of symptoms related to depression, anxiety, and stress. To measure negative emotional states of depression, anxiety and stress, a validated 21-item (DASS-21) was used.

Scores on each item ranged from 0 (*did not apply to me at all*) to 3 (*applied to me very much of the time*), indicating no symptomatology, to severe levels of symptomatology. The total scores for each of the negative emotional states were calculated separately by adding up the scores for the individual items and multiplying them by two to obtain the final scores. Higher scores indicated more depressive, anxiety and stress symptoms. Previous studies using the DASS-21 among a Ghanaian sample found the scale to be reliable ranging from 0.78 to 0.925.^21^ ^22^

### Statistical analysis

Statistical analysis was done using SPSS (v. 25). Categorical variables (i.e., age group, level of education, occupation, religion) were presented as frequencies and percentages. Means and standard deviations were calculated for age and duration of infertility. Depression, Anxiety and Stress Scores for age and duration of infertility were presented as Mean ±Standard deviation (SD) and analyzed using One-way ANOVA, followed by Bonferroni's post-hoc test to detect the true difference between groups after a significance is found. The multiple risk of outcome in one group compared to the other was measured using Relative Risk.

### Ethical approval

Ethical clearance was obtained from the institutional review board of the University of Cape Coast (Ref No: UC-CIRB/EXT/2019), and the ethical review board of the Cape Coast Teaching Hospital (Ref No: CCTHERC/EC/2019). Informed written consent was obtained from all the patients after explaining the objective of the study to them before recruitment into the study.

## Results

### Socio-demographic data of respondents

Table 1 shows the socio-demographic data of study respondents. With regard to age of female respondents, the majority (81.7%) were between the ages of 26-35 years. Most of the respondents (60.6%) had experienced infertility between 1-3 years. The majority of respondents (36.6%) had senior high school education, whilst 31% had tertiary education, followed by 25.4% with JHS education. Forty nine percent of respondents were traders, followed by teachers (15.5%), professionals (11.3%), and seamstresses (9.9%).

**Table 1:** Socio-demographic data of respondents

	Females (n=71)	Males (n=49)
**Age groups**	*n(%)*	*n(%)*
**21-25**	6 (8.5)	
**26-30**	31(43.7)	22(44.9)
**31-35**	27(38)	17(34.7)
≥**36**	7(9.9)	10(20.4)
**Total**	**71(100)**	**49(100)**
**1-3yrs**	43(60.6)	36(73.5)
**Duration of infertility**		
**4-6yrs**	23(32.4)	10(20.4)
**7yrs or more**	5(7)	3(6.1)
**Total**	**71(100)**	**49(100)**
**Education**		
**primary**	5(7)	8(16.3)
**JHS**	18(25.4)	4(8.2)
**SHS**	26(36.6)	5(10.2)
**Tertiary**	22(31)	32(65.3)
**Total**	**71(100)**	**49(100)**
**Religion**		
**catholic**	17(23.9)	5(10.2)
**Pentecostal**	36(50.7)	33(67.3)
**protestant**	13(18.3)	2(4.1)
**Muslim**	3(4.2)	7(14.3)
**other**	2(2.8)	2(4.1)
**Total**	**71(100)**	**49(100)**
**Occupation**		
**Trading**	35(49.3)	7(14.3)
**Professional**	8(11.3)	12(24.5)
**Teaching**	11(15.5)	3(6.1)
**Farming**	5(7)	8(16.3)
**Seamstress/tailor**	7(9.9)	1(2)
**Driving**		4(8.2)
**Unemployed**	4(56)	7(14.3)
**Other**	1(1.4)	7(14.3)
**Total**	**71(100)**	**49(100)**

*Professionals include health workers, security officers, accountants, administrators.

### Levels of depression, anxiety, and stress among the couples

Table 2 depicts the levels of depression, anxiety, and stress experienced by respondents after one year of unsuccessful drug therapy for infertility. There was no significant difference in the level of depression and stress among respondents (*p*=0.43, *RR*=1.15). Anxiety was the most prevalent psychological disorder experienced by respondents. This was statistically significant when compared to depression (*p* = 0.0096 **, *RR*= 1.40) and stress (*p*= 0.0005***, *RR*=1.62).

**Table 2:** Level of depression, anxiety and stress among respondents

	All	Females	Males
	*n(%)*	*n(%)*	*n(%)*
**Depression**			
**Normal**	68(56.7)	42(59.2)	26(53.1)
**Mild**	4(3.3)	3(4.2)	1(2)
**Moderate**	22(18.3)	14(19.7)	8(16.3)
**Severe**	20(16.7)	12(16.9)	8(16.3)
**Extremely severe**	6(5)	-	6(12.2)
**Total**	**120(100)**	**71(100)**	**49(100)**
**Anxiety**			
**Normal**	47(39.2)	27(38)	20(40.8)
**Mild**	31(25.8)	19(26.8)	12(24.5)
**Moderate**	4(3.3)	1(1.4)	3(6.1)
**Severe**	-	-	-
**Extremely severe**	38(31.7)	24(33.8)	14(28.6)
**Total**	**120(120)**	**71(100)**	**49(100)**
**Stress**			
**Normal**	75(62.5)	45(63.4)	30(61.2)
**Mild**	9(7.5)	3(4.2)	6(12.2)
**Moderate**	28(23.3)	16(22.5)	12(24.5)
**Severe**	8(6.7)	7(9.9)	1(2)
**Extremely severe**	-	-	-
**Total**	**120**	**71(100)**	**49(100)**

*All-means all respondents (n=120); female respondents (n=71); male respondents (n=49).

Over 43.3% of respondents experienced some form of depression. Severe and extremely severe depression affected about 21.7% of all respondents. When comparing gender and depression, men reported higher depressive symptoms compared to women (46.9 vs. 40.8%), although not statistically significant. Furthermore, men experienced severe and extremely severe forms of depression compared to women (28.6 vs16.9%; *p*= 0.1758, *RR*= 1.69). As shown in Table 2, extremely severe depressive symptoms was recorded only among male respondents. Table 2 shows that approximately two-thirds of all respondents (60.8%) exhibited some form of anxiety. More importantly, over 31.7% of respondents reported experiencing extremely severe anxiety after one-year of unsuccessful pharmacotherapy for infertility.

However, there was little gender disparity in relation to anxiety between females and males (61.9 vs. 59.1%), respectively. Anxiety and extremely severe anxiety were also nearly equal amongst females and males.

Stress was the least common psychological disorder experienced by respondents. Nonetheless, 37.5 percent of all respondents reported experiencing some form of stress associated with treatment failure. Compared to anxiety and depression, stress was predominantly reported to range from mild to moderate, with only 6.7 percent reporting severe stress. None of the respondents experienced extremely severe symptoms of stress. Nearly equal proportions of females and males experienced stress (36.6 vs. 38.8 %: *p* = 0.8494, *RR* = 1.059). However, severe stress was more common amongst women than men (9.9 vs. 2%, *p* = 0.1394, *RR*= 4.831) (Table 2).

### The effect of age and gender on psychological distress among respondents

Table 3 shows the effect of age and gender on psychological distress amongst respondents. Among the male respondents, the results indicated that age had a statistically significant effect on the symptoms of depression (*p*=0.041), anxiety (*p*<0.001), and stress (*p*=0.042) scores. As age increased, all three psychological distress increased. Respondents aged 26-30 years had significantly higher anxiety scores compared to respondents aged 30 years and above. Similarly, the same demography of men (26-30) reported statistically significant symptoms of depression and stress when compared to those between 31 and 35 years of age.

**Table 3:** Effect of age on psychological distress among respondents

			Males			Post-hoc statistics	
	Age categories	*n(%)*	Mean±SD	*F*	*p* value	age comparisons	*p* value
**Depression**	26-30	22(44.9)	8±5.84	3.43	0.041*	26-30*31-35	0.06
	31-35	17(34.69)	4.11±3.55			26-30*≥36	0.21
	>36	10(20.41)	4.5±5.02			31-35*≥36	1
	**Total**	**49(100)**	**5.94±5.23**				
**Anxiety**	26-30	22(44.9)	8.36±4.03	17.9	<0.001*	26-30*31-35	<0.001*
	31-35	17(34.69)	2.76±2.51			26-30*≥36	<0.001*
	>36	10(20.41)	3.1±1.79			31-35*≥36	1
	**Total**	**49(100)**	**5.35±4.17**				
**Stress**	26-30	22(44.9)	7.59±4.25	3.4	0.042*	26-30*31-35	0.18
	31-35	17(34.69)	5.06±4.26			26-30*≥36	0.06
	>36	10(20.41)	3.9±3.45			31-35*≥36	1
	**Total**	**49(100)**	**5.96±4.31**				
			**Females**				
**Depression**	20-25	6(8.45)	3±4.65	3.94	0.01*	21-25*26-30	0.55
	26-30	31(43.66)	6.29±5.09			21-25*31-35	0.85
	31-35	27(38.03)	5.89±3.67			21-25*≥36	1
	>36	7(9.86)	0.71±0.95			26-30*31-35	1
	**Total**	**71(100)**	**5.31±4.57**			26-30*≥36	0.02*
						31-35*≥36	0.04*
**Anxiety**	20-25	6(8.45)	3.33±2.58	2.59	0.06	21-25*26-30	0.99
	26-30	31(43.66)	6.03±4.82			21-25*31-35	0.71
	31-35	27(38.03)	6.41±4.33			21-25*>36	1
	>36	7(9.86)	2±2.24			26-30*31-35	1
	**Total**	**71(100)**	**5.55±4.46**			26-30*≥36	0.17
						31-35*≥36	0.11
**Stress**	20-25	6(8.45)	3±2.68	4.32	0.01*	21-25*26-30	0.39
	26-30	31(43.66)	6.77±4.63			21-25*31-35	0.13
	31-35	27(38.03)	7.81±5.01			21-25*≥36	1
	>36	7(9.86)	2±2.16			26-30*31-35	1
	**Total**	**71(100)**	**6.38±4.81**			26-30*≥36	0.08
						31-35*≥36	0.02*

*Means p-value is significant at 0.05 level. Statistical analysis was done using one-way ANOVA, and post-hoc analysis was done using Bonferroni's test. * means p < 0.05.

### The effect of the duration of infertility on psychological distress among respondents

Table 4 shows the effect of the duration of infertility on psychological distress among respondents. The results indicated that, the duration of infertility significantly affected anxiety levels (*p*=0.01, *F*=4.55), but showed no significant effect on depression (*p*=0.51, *F*=0.68) and stress (*p*=0.06, *F*=2.92) levels. Approximately 66.7% of respondents who experienced these psychological symptoms had suffered infertility over 1-3 years. Furthermore, post-hoc analysis using Bonferroni tests indicated that, respondents with a duration of 4 to 6 years of infertility differed significantly in psychological distress scores from those with 7 years and above. No gender disparity was noted with respect to the effect of duration of infertility on psychological distress among infertile couples.

**Table 4:** Effect of duration of infertility on psychological distress among respondents

	All respondents			
	Duration (years)	*n(%)*	Mean±SD	*p* value
**Depression**	1 - 3	79(65.83)	5.82±4.99	0.51
	4 - 6	33(27.5)	4.76±4.73	
	≥7	8(6.67)	6.38±3.62	
	**Total**	**120(100)**	**5.57±4.84**	
**Anxiety**	1 - 3	79(65.83)	5.86±4.37	0.013*
	4 - 6	33(27.5)	3.85±3.85	
	>7	8(6.67)	8.25±3.81	
	**total**	**120(100)**	**5.47±4.33**	
**Stress**	1 - 3	79(65.83)	6.76±4.81	0.06
	4 - 6	33(27.5)	4.60±4.0	
	≥7	8(6.67)	7.375±3.33	
	**total**	**120(100)**	**6.21±4.60**	
		**Males**		
**Depression**	1 - 3	36(73.47)	2.58±1.63	0.11
	4 - 6	10(20.41)	1.80±1.14	
	≥7	3(6.12)	1.0±0.00	
	**Total**	**49(100)**	**2.33±1.55**	
**Anxiety**	1 - 3	36(73.47)	2.81±1.75	0.12
	4 - 6	10(20.41)	1.70±1.34	
	≥7	3(6.12)	1.67±0.58	
	**Total**	**49(100)**	**2.51±1.68**	
**Stress**	1 - 3	36(73.47)	1.83±0.97	0.08
	4 - 6	10(20.41)	1.10±0.32	
	≥7	3(6.12)	1.67±1.15	
	**Total**	**49(100)**	**1.67±0.92**	
		**Females**		
**Depression**	1 - 3	43(60.56)	5.23±4.35	0.15
	4 - 6	23(32.39)	4.65±5.17	
	≥7	5(7.04)	9.00±0.00	
	**Total**	**71(100)**	**5.31±4.52**	
**Anxiety**	1 - 3	43(60.56)	5.34±4.53	0.09
	4 - 6	23(32.39)	4.39±4.00	
	≥7	5(7.04)	11±0.00	
	**Total**	**71(100)**	**5.55±4.46**	
**Stress**	1 - 3	43(60.56)	6.68±5.13	0.32
	4 - 6	23(32.39)	4.91±4.37
	≥7	5(7.04)	9.00±0.00
	**Total**	**71(100)**	**6.38±4.81**

* Means p-value is significant at 0.05 level. Statistical analysis was done using one-way ANOVA followed by Bonferroni's post hoc.

Generally, among female respondents, psychological distress scores for depression, anxiety and stress increased with age from 21 to 35. After 35years, there was a decline in the psychological distress scores. This was statistically significant for depression (*p*=0.01) and stress (*p*=0.01) levels but not anxiety (*p*=0.06). Furthermore, a post hoc Bonferroni analysis to examine the mean differences for the psychological distress based on respondents ages showed the following results. 26-30years and ≥36years (*p*=0.02), and aged 31-35years and ≥36years (*p*=0.04). The post-hoc test on stress level also showed a significant difference between the ages 31-35years and ≥36years (*p* = 0.02).

## Discussion

The levels of depression, anxiety and stress relied upon respondents' own subjective information, and this may lead to biased report. Second, the study was a cross-sectional survey and hence we could not attribute causality to our results. Also, confounders such as the non-adherence to treatment could not absolutely be ruled out on the part of the respondents. Additionally, our sample were recruited from a clinic, hence these results may not be applicable to a non-clinical sample. There might be other unexamined factors which may obfuscate the results of this studies; hence implications and applicability of these findings should be done with caution. Many risk factors for development of psychological distress among infertile couples such as cultural and personality factors, and social support were not assessed in this study.

The study was conducted to assess the level of psychological distress among infertile couples after one year of unsuccessful drug treatment, and to evaluate gender differences in the experience of psychological symptoms. The heterogeneous socio-demography among the respondents in the study reflected the diverse socioeconomic and cultural plurality of the population of the Central Region of Ghana. Although socio-demographic factors are known to influence psychological status, the level of this association is inconclusive in current literature. In Ghana, children are highly esteemed for social-cultural, religious and economic reasons^12^ and as such, there is the motivation to seek early treatment for infertility. Childless couples experience feelings of dejection, inadequacy and guilt. Such negative experiences are compounded by the pressure mounted on the couple by both families, especially that of the male partner. 23

Childbearing is seen as a continuation of family lineage in many Ghanaian societies. Customarily, the new couple are expected to bring forth a baby within at least one year of marriage, and anything short of that is unacceptable. Most of the blame is shifted on the women since they are regarded as the cause of infertility, even when the etiology is not associated with the woman. The tendency of the society to blame the woman leads to unimagined psychological dilemmas, fear of divorce, abandonment and polygamy.^23^,^24^ The men are usually stigmatized, verbally abused, and not accorded any social roles within the society due to infertility. 24 Such expectations of the society from the struggling couple tend to add more woes to their sorrows.

It was evident from the study that respondents experienced diverse symptoms of depression, anxiety, and stress. Negative psychosocial distress has been implicated to be a potential cause as well as a consequence of infertility 25 Furthermore, infertility treatment may be associated with negative psychological symptoms.^6^ Treatment of infertility is associated with higher depressive symptoms. Depressive symptoms include a lack of interest or a perceived sense of loss, sadness, lack of control over a situation or event. Over 43.3% of respondents in this study experienced some form of depression. Severe and extremely severe depression affected about 21.7% of all respondents. In a systematic review and meta-analysis of depression in infertility, the prevalence among infertile women in low and middle income countries was estimated to be 44.32%.^24^ Depression during infertility appears to be strongly influenced by the economic status of the society. Consequently, lower prevalence rates have been reported in high income countries.^26^ Most studies report that infertile women experience higher levels of depression than infertile men. 27In addition, infertile men suffering from depression adopt coping strategies such as distancing, self-control, escape-avoidance and painful problem-solving whereas infertile women typically adopt confrontive coping and social support-seeking behaviors. In this study it was noted that depression was predominantly higher in men than in women (46.9 vs. 40.8%) and men suffered more severe and extremely severe forms of depression than women. In fact, extremely severe depression was only recorded in male respondents. A possible reason for this divergence in findings could be due to the timing of the assessment. Most studies measure depression and other psychological distress prior to initiation of therapy and/or during therapy.

However, this study was a post-therapy assessment. Furthermore, sociocultural differences can also account for this difference. Many of the studies on depression in infertile men were done in Western populations. Culturally, it is very ‘unmanly’ for a Ghanaian man to vocalize his feelings or failures. Consequently, Ghanaian men may likely adopt maladaptive coping strategies like escape-avoidance and less social support-seeking behaviors after unsuccessful pharmacotherapy of infertility. This behavior can easily precipitate negative depressive symptoms and cause extremely severe depression in infertile men.

In the current study, most respondents (60.8%) reported anxiety as the predominant psychological distress symptoms. Anxiety presents as a perceived sense of threat, tension and irrational worry. This study did not determine anxiety levels before initiation of pharmacotherapy. It has been reported that infertile women have higher anxiety scores than fertile women. 28In this study, there was no gender difference in the levels and severity of anxiety among infertile couples.

However, unlike depression, a study among infertile women undergoing assisted reproductive treatment showed that anxiety levels did not significantly change prior to treatment and also after unsuccessful treatment. 26It is very possible that the high levels of anxiety noted among respondents in this study could have been a motivating factor for the treatment-seeking behaviors of young couples.

Stress but not depression or anxiety was the least self-reported psychological distress experienced by the respondents and this was segregated based on gender, years of marriage/infertility. Nonetheless, 37.5% of all respondents reported experiencing some form of stress associated with treatment failure. Generally, respondents experienced mild to moderate symptoms of stress. The prevalence of stress among infertile women varies across cultures. It has been argued that a major cause of infertility-associated stress in Ghanaian women is stigma. 3 Social support and education level are negatively associated with stress.

Nearly equal proportions of females and males experienced stress. However, severe stress was significantly more common among women than men. According to the literature, infertility stress triggers are stronger for women than men. This is supported by a study by Cserepes, et al. 29 that indicates that regardless of who is infertile among the couple, infertility is considered a stressful situation for the woman. In a pro-natal society like Ghana, most women may see infertility as role failure and personal shortfall; this creates feelings of guilt which are compounded by societal stigma. More importantly, chronic stressors can place a person at higher risk for major depressive disorder than the acute nature of many events in life.

In the current study, age and duration of infertility had effect on the level of psychological distress experienced by respondents. The findings from this study indicates that the psychological distress symptoms were more common among female respondents aged 26-35 years, which is consistent with other studies.^12^, ^28^ According to Noorbala, et al. 28 better coping mechanisms among older infertile couples might result in less depressive symptoms. Moreover, the knowledge that fertility status, particularly for the woman, declines with age may put the couple under an intense psychological dilemma, which may probably contribute to the high level of these emotional experiences as identified in the current study.^12^

Duration of infertility also had effect on symptoms of psychological distress such that couples who had experienced infertility for seven years and above exhibited increased levels of psychological distress, especially anxiety symptoms.

Similar observations have established that increased periods of infertility correlate with increasing psychological distress. 29 During the initial phase of infertility diagnosis, the couple have higher expectations of positive treatment outcome. However, such hopes tend to dwindle with cycles of unsuccessful treatment, leading to increased risk and burden of depression, anxiety and stress symptoms.^12^

These psychological symptoms, aside from being major consequences of infertility, may also play a crucial role in the etiology of infertility. 25However, in most African societies, these psychological symptoms are not assessed to receive the needed treatment, a factor that might contribute to apathy and a lack of interest in seeking further treatment and impact treatment success subsequently. Although the burden of seeking infertility care is heavily placed on women, especially in Africa, such findings should not downplay the fact that, infertile men also stand an equal chance of suffering these psychological symptoms, and sometimes to a higher level compared to women. Hence, fertility counselors must pay equal attention to both partners during the counseling process to help prevent any consequences that might emanate from these uncomfortable experiences. Psychotherapy may be essential in subfertility treatment as to mitigate some of these negative experiences with particular focus on the age of the couples and duration of infertility.

## Conclusion

Unsuccessful pharmacotherapy of infertility is associated with varied degrees of psychological distress which can be affected by age, duration of infertility and gender. Anxiety was found to be the leading psychological distress among the couple, followed by depression and stress. Psychotherapy and counseling services should be made available to infertile couples after an unsuccessful treatment to mitigate the impact of psychological distress on subsequent treatment.
